# Supplementary material for: Subsistence strategy mediates ecological drivers of human violence
Source: PLoS One. 2022 May 23;17(5):e0268257. doi: 10.1371/journal.pone.0268257 (PMC9126380; doi:10.1371/journal.pone.0268257)
Supplement: S2 File — (PDF) [file pone.0268257.s002.pdf]

Overview
R Preamble
Primary Data
Test for Correlation
Model Formulas
Exploratory Models
Explanatory Models
Quasi-binomial Models
ANOVA
Residual Autocorrelation
Results
Session Info
Appendix: Plotting Functions
References

Supplement A

13 December, 2021

Overview

Goal: to evaluate ecological drivers of violence across subsistence economies.

Data: estimates of proportional violence (Sigma) among ethnographic and archaeological populations, along with the mean (NPP) and standard deviation (NPPsd) of net primary productivity.

Method: quasibinomial generalized linear model

R Preamble

```
library(broom)
library(dplyr)
library(ggplot2)
library(here)
library(kableExtra)
library(patchwork)
library(rnaturalearth)
library(sf)
library(spdep)
library(tibble)
library(tidyr)
library(viridis)
```

Miscellaneous

```
# functions for collecting data to plot models
# also some stuff to print the session info in an html table
source(here("R/utils.R"))

# set seed to today
today <- 201020

set.seed(today)
```

Primary Data

Our response variable is proportional violence. In the archaeological case, that means proportion of osteological samples; in the ethnographic case, proportion of population estimates.

The predictor variables are net primary productivity, both the mean and standard deviation. These are extracted using the `extract()` function from the `raster` package with a 50-kilometer buffer. Note that we also use subsistence economy as a factor level interaction term.

```
violence_data <-
  here("data", "EthnoArch_DF_Complete.csv") %>%
  read.csv() %>%
  mutate(MoS = case_when(MoS == "F" ~ "Foraging",
                         MoS == "H" ~ "Horticulture",
                         MoS == "A" ~ "Farming",
                         TRUE ~ MoS)) %>%
  select(-popdens, -vd_trauma)
```

We downscale NPP and NPPsd by four orders of magnitude as per the MODIS documentation (see *MOD17A3_data_info.txt*). The unit of measure is thus $kgC/m^2/year$.

```
violence_data <-
  violence_data %>%
  mutate(NPP = (NPP / 10000),
         NPPsd = (NPPsd / 10000))
```

Region	Soc	MoS	complexity	data_type	Sigma	NPP	NPPsd	Note
S_Amer	Ache	Foraging	Egalitarian	Ethno	0.4300	0.9944304	0.2048640	E. Paraguay
S_Amer	Achuar	Horticulture	Egalitarian	Ethno	0.4200	1.5986696	0.2653122	
Australia	Anbara	Foraging	Egalitarian	Ethno	0.0400	0.3331933	0.2509622	N. Australia
NA	Blackfoot	Foraging	Egalitarian	Ethno	0.3300	0.2759457	0.0445140	Plain Indians; 1858 CE
SE_Asia	Casiguran Agta	Foraging	Egalitarian	Ethno	0.1200	0.7810883	0.5184520	Philippines; 1962 - 1977 CE
PNG	Dugum Dani	Horticulture	Egalitarian	Ethno	0.1550	1.1975081	0.3710800	Grand Valley Dani
PNG	Enga	Horticulture	Egalitarian	Ethno	0.3480	1.4668741	0.2952693	Papua New Guinea - all of Enga, but datum is same as Mae Enga
PNG	Gebusi	Horticulture	Egalitarian	Ethno	0.3270	1.5405185	0.0637542	Papua New Guinea

Society Map

To give a sense of the spatial distribution of these archaeological and ethnographic populations, we provide a world map.

```
societies <-
  violence_data %>%
  st_as_sf(coords = c("X", "Y"), crs = 4326)

world <- ne_countries(scale = "medium", returnclass = "sf")
```

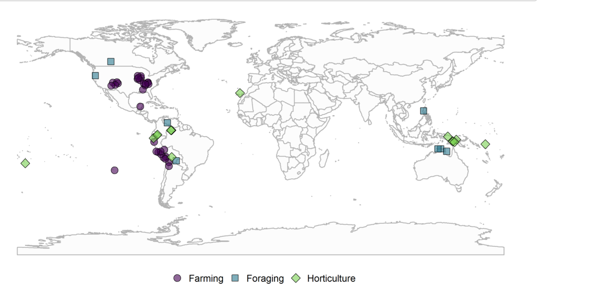

2d Density

The density of societies along the gradient of NPP (mu-by-sd) for these populations should provide some sense of the ecological niche they occupy, though this is unweighted by the global prevalence of these conditions.

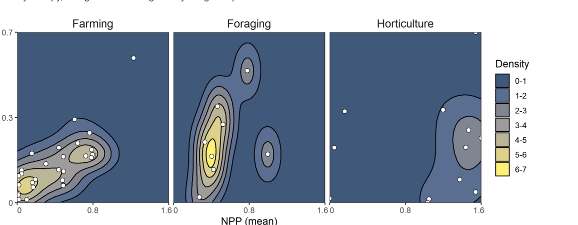

Test for Correlation

A test of correlation in predictors and of autocorrelation in the response should be a required component of any likelihood estimation.

Predictors: *Pearson correlation*

```
with(violence_data, cor.test(NPP, NPPsd))
```

```
##
##  Pearson's product-moment correlation
##
## data:  NPP and NPPsd
## t = 3.7183, df = 51, p-value = 0.0004997
## alternative hypothesis: true correlation is not equal to 0
## 95 percent confidence interval:
##  0.2188434 0.6508676
## sample estimates:
##       cor
## 0.4618196
```

Response: *Moran's I*

Now we check for spatial autocorrelation in our response variable, Sigma (i.e., proportion of violence), using a Monte Carlo simulation of the Moran's I test, which is implemented with the `moran.mc()` function from the `spdep` package. The null hypothesis for this test is that there is no significant spatial autocorrelation in the response. To build a neighborhood matrix, we use a simple inverse distance weighting.

We want to test for spatial autocorrelation across all societies taken together and within groups of societies defined by mode of subsistence, so we define a function `get_moran` to execute this procedure while satisfying the DRY principle.

```
get_moran <- function(x, variable){

  coords <- st_coordinates(x)

  dw <- as.matrix(dist(coords))

  idw <- 1/dw

  diag(idw) <- 0

  idw[is.infinite(idw)] <- 0

  swm_listw <- mat2listw(idw)

  df <- st_drop_geometry(x)

  mi_test <- moran.mc(df[, variable, drop = TRUE],
                      listw = swm_listw,
                      nsim = 999)

  df <- data.frame(
    variable = variable,
    statistic = round(mi_test$statistic, digits = 3),
    rank = as.integer(mi_test$parameter),
    pval = round(mi_test$p.value, digits = 3)
  )

  rownames(df) <- NULL

  return(df)

}
```

```
all_socs <-
  societies %>%
  get_moran("Sigma") %>%
  mutate(MoS = "All")

moran_table <-
  societies %>%
  group_by(MoS) %>%
  nest() %>%
  mutate(Moran = lapply(data, get_moran, variable = "Sigma")) %>%
  unnest(Moran) %>%
  select(-data)

moran_table <-
  moran_table %>%
  bind_rows(all_socs, .) %>%
  select(MoS, everything())

rm(all_socs)
```

Moran's I Test
Alternative: no spatial autocorrelation.

MoS	variable	statistic	rank	pval
All	Sigma	0.366	970	0.030
Foraging	Sigma	-0.378	337	0.663
Horticulture	Sigma	0.068	705	0.295
Farming	Sigma	0.394	955	0.045

It appears that farming populations are almost entirely responsible for spatial autocorrelation in the response.

Model Formulas

Here are our model specifications.

```
fm_nul <- Sigma ~ 1

fm_bas <- Sigma ~ (NPP + NPPsd)

fm_MoS <- Sigma ~ (NPP + NPPsd) * MoS

fm_typ <- Sigma ~ (NPP + NPPsd) * data_type
```

where

- Sigma = proportion of violence (0 to 1)
- NPP = net primary productivity mean
- NPPsd = net primary productivity standard deviation
- MoS = factor, mode of subsistence (foraging, farming, horticulture)
- data_type = population sample (ethnographic or archaeological)

Exploratory Models

Binomial Model

First, a test for scaled dispersion. In a binomial distribution, the variance should equal the mean. Under-dispersion suggests $variance < mean$; over-dispersion $variance > mean$. We can test for this using a χ^2 comparing the deviance of a binomial model to its residual degrees of freedom. This will tell us whether we should use a quasibinomial model.

```
mod_binom <- glm(fm_MoS,
                 family = binomial,
                 data = violence_data)
```

```
pchisq(
  deviance(mod_binom),
  df.residual(mod_binom),
  lower.tail = TRUE
)
```

```
## [1] 1.876657e-17
```

Rejection of the null hypothesis in a lower.tail test indicates under-dispersion, suggesting that we need to use a quasibinomial error distribution in our model.

Quasi-binomial Model – Data Type

Here we include an interactive data type to test for differences in ethnographic and archaeological data.

```
mod_typ <- glm(fm_typ,
               family = quasibinomial,
               data = violence_data)
```

Summary Fit

null.deviance	df.null	logLik	AIC	BIC	deviance	df.residual	nobs	dispersion
5.979	52	NA	NA	NA	3.963	47	53	0.084

Coefficient Estimates

term	estimate	std.error	statistic	p.value
(Intercept)	-1.736	0.250	-6.945	0.000
NPP	-1.681	0.572	-2.940	0.005
NPPsd	3.716	1.590	2.336	0.024
data_typeEthno	0.194	0.530	0.366	0.716
NPP:data_typeEthno	2.519	0.676	3.724	0.001
NPPsd:data_typeEthno	-6.185	1.935	-3.197	0.002

This is suggestive, but we believe it is driven almost entirely by the fact that ethnographic data sets consist primarily of foraging populations, archaeological data sets of farming populations.

```
tab <- with(violence_data, table(MoS, data_type))
```

	Arch	Ethno
Farming	31	0
Foraging	0	8
Horticulture	3	11

```
chi <- chisq.test(tab)
```

statistic	42.75
p.value	0
parameter	2
method	Pearson's Chi-squared test

A significant Chi squared test indicates that the distribution of mode of subsistence over data types is non-random.

Explanatory Models

Quasi-binomial Models

As a baseline for evaluating the significance of mode of subsistence, we first fit an intercept only or null model and a model with no test for interaction with MoS. Then we test for interaction in mode of subsistence.

```
mod_nul <- glm(fm_nul,
               family = quasibinomial,
               data = violence_data)

mod_bas <- glm(fm_bas,
               family = quasibinomial,
               data = violence_data)

mod_MoS <- glm(fm_MoS,
               family = quasibinomial,
               data = violence_data)
```

Model statistics

model	null.deviance	df.null	logLik	AIC	BIC	deviance	df.residual	nobs
Null	5.9791	52	NA	NA	NA	5.9791	52	53
Base	5.9791	52	NA	NA	NA	5.5138	50	53
MoS	5.9791	52	NA	NA	NA	3.3844	44	53

Coefficient estimates

	estimate	std.error	statistic	p.value
**Null**				
(Intercept)	-1.5041	0.1202	-12.5162	0.0000
**Base**				
(Intercept)	-1.5696	0.2239	-7.0117	0.0000
NPP	0.5134	0.2526	2.0322	0.0475
NPPsd	-1.3545	0.9895	-1.3689	0.1771
**MoS**				
(Intercept)	-1.7953	0.2448	-7.3344	0.0000
NPP	-2.7815	0.7370	-3.7740	0.0005
NPPsd	6.9563	2.1135	3.2913	0.0020
MoSForaging	-0.0017	0.7239	-0.0024	0.9981
MoSHorticulture	0.1434	0.5506	0.2604	0.7957
NPP:MoSForaging	5.2511	1.2849	4.0868	0.0002
NPP:MoSHorticulture	3.5463	0.8256	4.2954	0.0001
NPPsd:MoSForaging	-11.2655	3.0299	-3.7181	0.0006
NPPsd:MoSHorticulture	-8.7846	2.4024	-3.6565	0.0007

A low dispersion value indicates under-dispersion, which the quasi-binomial model should account for.

ANOVA

```
anova(mod_nul, mod_bas, mod_MoS, test = "LRT")
```

```
## Analysis of Deviance Table
##
## Model 1: Sigma ~ 1
## Model 2: Sigma ~ (NPP + NPPsd)
## Model 3: Sigma ~ (NPP + NPPsd) * MoS
##   Resid. Df Resid. Dev Df Deviance Pr(>Chi)
## 1        52     5.9791
## 2        50     5.5138  2  0.46528  0.04582 *
## 3        44     3.3844  6  2.12939 8.54e-05 ***
## ---
## Signif. codes:  0 '***' 0.001 '**' 0.01 '*' 0.05 '.' 0.1 ' ' 1
```

As we expected, mode of subsistence makes a significant contribution to model fit.

Residual Autocorrelation

```
# get the raw residuals
societies$residuals <- residuals(mod_MoS, type = "response")

mi_res <- get_moran(societies, "residuals")
```

Moran's I Test
Alternative: no spatial autocorrelation.

variable	statistic	rank	pval
residuals	0.169	836	0.164

Spatial autocorrelation is largely gone from the response once fitted to the full model. This suggests that one of our predictor variables has captured any latent spatial process in the response. This is likely owing to the use of mode of subsistence as an interaction term. See, for example, the world map above.

Results

Here we provide two visual representations of our results. The marginal response shows change in the response for a selected variable while holding all other variables at their mean, for each mode of subsistence. What we are calling the "punnett response" shows change in the response within the state space defined by our two primary variables (NPP mu and sd). The latter is more indicative of our overarching theoretical expectations.

Marginal Response

```
new_data <-
  with(violence_data,
       data.frame(
         NPP = seq(min(NPP), max(NPP), length.out = 200),
         NPPsd = seq(min(NPPsd), max(NPPsd), length.out = 200)
       )
  )

margins_all <-
  rbind(
    get_margins(mod_bas, new_data, "NPP"),
    get_margins(mod_bas, new_data, "NPPsd")
  )

MoS <- c("Farming", "Foraging", "Horticulture")

margins_mos <-
  lapply(MoS, function(z){

    new_data$MoS <- z

    # Farming is the reference class
    if (z == "Farming") {

      vnpp   <- "NPP"
      vnppsd <- "NPPsd"

    } else {

      vnpp  <- paste0("NPP:MoS", z)
      vnppsd <- paste0("NPPsd:MoS", z)

    }

    df <-
      rbind(
        get_margins(mod_MoS, new_data, vnpp),
        get_margins(mod_MoS, new_data, vnppsd)
      )

    df <- cbind("MoS" = z, df)

    return(df)

  })

margins_mos <- do.call(rbind, margins_mos)
```

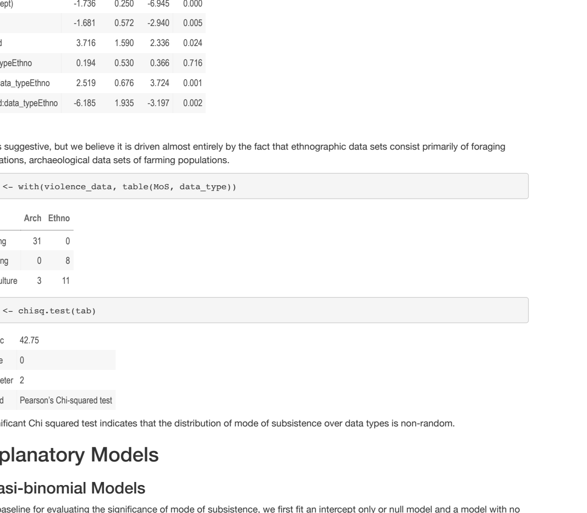

Note that the dashed line represents the strategy-insensitive model.

Punnett Response

```
punnett_all <- get_punnett(mod_bas)

punnett_all$MoS <- "All"

punnett_MoS <-
  lapply(MoS,
         function(z) {

           df <- get_punnett(mod_MoS, MoS = z)

           df$MoS <- z

           return(df)

         })

punnett_MoS <- do.call(rbind, punnett_MoS)
```

The punnett for the base model is just a dark blue-purple rectangle.

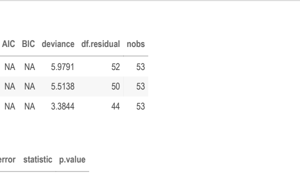

And here are the punnetts by mode of subsistence.

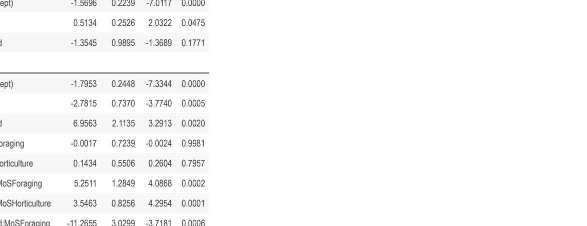

Session Info

R Version: R version 4.1.0 (2021-05-18)
Nickname: Camp Pontanezen
Operating System: Windows 10 x64 (build 19043)

Package	Version	Maintainer	URL
broom	0.7.10	Simon Couch	https://broom.tidymodels.org/ https://github.com/tidymodels/broom
dplyr	1.0.7	Hadley Wickham	https://dplyr.tidyverse.org https://github.com/tidyverse/dplyr
ggplot2	3.3.5	Thomas Lin Pedersen	https://ggplot2.tidyverse.org https://github.com/tidyverse/ggplot2
here	1.0.1	Kirill Müller	https://here.r-lib.org/ https://github.com/r-lib/here
kableExtra	1.3.4.9000	Hao Zhu	http://haozhu233.github.io/kableExtra/ https://github.com/haozhu233/kableExtra
patchwork	1.1.1	Thomas Lin Pedersen	https://patchwork.data-imaginist.com https://github.com/thomasp85/patchwork
rnaturalearth	0.1.0	Andy South	https://github.com/ropenscilabs/rnaturalearth
sf	1.0-4	Edzer Pebesma	https://r-spatial.github.io/sf/ https://github.com/r-spatial/sf/
sp	1.4-6	Edzer Pebesma	https://github.com/edzer/sp/ https://edzer.github.io/sp/
spData	2.0.1	Jakub Nowosad	https://nowosad.github.io/spData/
spdep	1.1-12	Roger Bivand	https://github.com/r-spatial/spdep/
tibble	3.1.6	Kirill Müller	https://tibble.tidyverse.org/ https://github.com/tidyverse/tibble
tidyr	1.1.4	Hadley Wickham	https://tidyr.tidyverse.org https://github.com/tidyverse/tidyr
viridis	0.6.2	Simon Garnier	https://sjmgarnier.github.io/viridis/ https://github.com/sjmgarnier/viridis/
viridisLite	0.4.0	Simon Garnier	https://sjmgarnier.github.io/viridisLite/ https://github.com/sjmgarnier/viridisLite/

Appendix: Plotting Functions

```
#' Get marginal response data
#'
#' @param x a model
#' @param new_data data.frame
#' @param variable character, the independent variable
#'
#' @return a data.frame with variable, x, y, confidence low, and confidence high
#'
get_margins <- function(x, new_data, variable){

  significant <- is_significant(x, variable)

  if (grepl("MoS", variable)) variable <- strsplit(variable, ":")[[1]][[1]]

  if (significant) {

    res <- estimate(x, new_data, variable)

  } else {

    res <- zero_out(variable, new_data)

  }

  return(res)

}

#' get coefficient pvalue and compare to alpha
#'
is_significant <- function(x, variable, alpha = 0.05) {

  p <-
    x %>%
    summary() %>%
    coefficients() %>%
    as.data.frame() %>%
    rownames_to_column(var = "variable") %>%
    filter(variable == !!variable) %>% # !! = unquote
    pull("Pr(>|t|)")

  p < alpha

}

#' return a zeroed-out dataframe for non-significant coefficients
#'
zero_out <- function(variable, new_data) {

  data.frame(
    variable = variable,
    x = new_data[, variable],
    y = 0,
    conf_hi = 0,
    conf_lo = 0
  )

}

#' predict with significant coefficient at margin or mean of other variables
#'
estimate <- function(x, new_data, variable) {

  # center alternate variable
  if (variable == "NPP") {

    new_data[, "NPPsd"] <- mean(x$data[, "NPPsd"])

  } else {

    new_data[, "NPP"] <- mean(x$data[, "NPP"])

  }

  # https://fromthebottomoftheheap.net/2018/12/10/confidence-intervals-for-glms/
  inv_link <- family(x)$linkinv

  y <- predict(x, newdata = new_data, se.fit = TRUE)

  data.frame(
    variable = variable,
    x        = new_data[, variable],
    y        = inv_link(y$fit),
    conf_hi  = inv_link(y$fit + (2 * y$se.fit)),
    conf_lo  = inv_link(y$fit - (2 * y$se.fit))
  )

}


#' predict at all combinations of independent variables
#'
get_punnett <- function(mod, n = 200, MoS = NULL){

  xrng <- range(mod$data[, "NPP"])
  yrng <- range(mod$data[, "NPPsd"])

  x <- seq(xrng[[1]], xrng[[2]], length.out = n)

  y <- seq(yrng[[1]], yrng[[2]], length.out = n)

  new_data <-
    expand.grid(
      x = x,
      y = y
    )

  names <- c("NPP", "NPPsd")

  if (!is.null(MoS)) {

    new_data$MoS <- MoS
```

```
    names <- c(names, "MoS")

  }

  names(new_data) <- names

  log_estimate <- predict(mod, newdata = new_data)

  inv_link <- family(mod)$linkinv

  estimate <- inv_link(log_estimate)

  cbind(new_data, "z" = estimate)

}
```

References

Andrushko, Valerie A., and Elva C. Torres. 2011. “Skeletal Evidence for Inca Warfare from the Cuzco Region of Peru.” *American Journal of Physical Anthropology* 146 (3): 361–72. https://doi.org/https://doi.org/10.1002/ajpa.21574.

Arkush, Elizabeth, and Tiffiny A. Tung. 2013. “Patterns of War in the Andes from the Archaic to the Late Horizon: Insights from Settlement Patterns and Cranial Trauma.” *Journal of Archaeological Research* 21 (4): 307–69. http://www.jstor.org/stable/42635586.

Blanchard, Lance. 2011. “Raised Walls and Broken Bones: An Analysis of Defensive Architecture and Violent Skeletal Trauma in Late Prehistoric Eastern North America.” PhD thesis, Department of Anthropology.

Bowles, Samuel. 2009. “Did Warfare Among Ancestral Hunter-Gatherers Affect the Evolution of Human Social Behaviors? On JSTOR.” *Science* 324 (5932): 1293–98. https://www.jstor.org/stable/20494089.

Cole, Sarah M. 2012. “Population Dynamics and Warfare in the Central Mesa Verde Region.” In *Emergence and Collapse of Early Villages: Models of Central Mesa Verde Archaeology*, edited by Timothy A. Kohler and Mark D. Varien, 197–218. University of California Press.

Darcy, K., C. M. Gaither, and K. Koschmeider. 2010. “Trauma Patterns of the Chachapoya.” In *50th Annual Meeting of the IAS*. Berkeley, CA.

Fouant, M. M. C. 1984. *The Skeletal Biology and Pathology of Pre-Columbian Indians from Northern Chile*. Richmond, VA: Virginia Commonwealth University.

Fry, D. P., ed. 2013. *War, Peace, and Human Nature: The Convergence of Evolutionary and Cultural Views.* Oxford University Press.

Gat, A. 2006. *War in Human Civilization*. Oxford University Press.

Harrod, R. P. 2013. “Chronologies of Pain and Power: Violence, Inequality, and Social Control Among Ancestral Pueblo Populations (AD 850-1300).” Doctoral {{Dissertation}}, Department of Anthropology: University of Nevada, Las Vegas.

Hill, K., and A. M. Hurtado. 2017. *Achè Life History: The Ecology and Demography of a Foraging People*. New York: Routledge.

Hill, Kim, A. M. Hurtado, and R. S. Walker. 2007. “High Adult Mortality Among Hiwi Hunter-Gatherers: Implications for Human Evolution.” *Journal of Human Evolution* 52 (4): 443–54. https://doi.org/10.1016/j.jhevol.2006.11.003.

Keeley, Lawrence H. 1996. *War Before Civilization*. New York, NY: Oxford University Press.

Kellner, C. M. 2002. “Coping with Environmental and Social Challenges in Prehistoric Peru: Bioarchaeological Analyses of Nasca Populations.” Doctoral {{Dissertation}}, Department of Anthropology: University of California, Santa Barbara.

Knauft, Bruce M., Martin Daly, Margo Wilson, Leland Donald, George E. E. Morren Jr., Keith F. Otterbein, Marc Howard Ross, H. U. E. Thoden van Velzen, and W. van Wetering. 1987. “Reconsidering Violence in Simple Human Societies: Homicide Among the Gebusi of New Guinea [and Comments and Reply].” *Current Anthropology* 28 (4): 457–500.

Kohler, Timothy A., Scott G. Ortman, Katie E. Grundtisch, Carly M. Fitzpatrick, and Sarah M. Cole. 2014. “The Better Angels of Their Nature: Declining Violence Through Time Among Prehispanic Farmers of the Pueblo Southwest.” *American Antiquity* 79 (3): 444–64. https://doi.org/10.7183/0002-7316.79.3.444.

Kurin, Danielle S. 2012. “The Bioarchaeology of Collapse: Ethnocide and Ethnogenesis in Post-Imperial Andahuaylas, Peru (AD 900-1200).” Doctoral {{Dissertation}}, Department of Anthropology: Vanderbilt University.

McCool, Weston C., Tiffiny A. Tung, Joan Brenner Coltrain, A. Accinelli, and Douglas J. Kennett. 2021. “The Character of Conflict: A Bioarchaeological Study of Violence in the Nasca Highlands of Peru During the Late Intermediate Period (950 1450 C.E.).” *American Journal of Physical Anthropology* in-press.

Milner, George R., Eve Anderson, and Virginia G. Smith. 1991. “Warfare in late prehistoric West-Central Illinois.” *American Antiquity* 56 (4): 581–603. https://doi.org/10.2307/281538.

Murphy, Melissa S., Catherine Gaither, Elena Goycochea, John W. Verano, and Guillermo Cock. 2010. “Violence and Weapon-Related Trauma at Puruchuco-Huaquerones, Peru.” *American Journal of Physical Anthropology* 142 (4): 636–49. https://doi.org/10.1002/ajpa.21291.

Nystrom, Kenneth C. 2004. “Trauma y Identidad Entre Los Chachapoya.” In *Proceedings of the Primera Conferencia Internacional Sobre El Arte, La Arqueología y La Etnohistoria de Los Chachapoya, Leymebamba, Perú*, 9:20–21. Peru: Sian.

Owens, L. S. 2007. “Craniofacial Trauma in the Prehispanic Canary Islands.” *International Journal of Osteoarchaeology* 17 (5): 465–78. https://doi.org/10.1002/oa.898.

Scott, Rachel M., and Hallie R. Buckley. 2010. “Biocultural Interpretations of Trauma in Two Prehistoric Pacific Island Populations from Papua New Guinea and the Solomon Islands.” *American Journal of Physical Anthropology* 142 (4): 509–18. https://doi.org/10.1002/ajpa.21250.

———. 2014. “Exploring Prehistoric Violence in Tonga: Understanding Skeletal Trauma from a Biocultural Perspective.” *Current Anthropology* 55 (3): 335–47. https://doi.org/10.1086/676477.

Serafin, Stanley, Carlos Peraza Lope, and Eunice Uc González. 2014. “Bioarchaeological Investigation of Ancient Maya Violence and Warfare in Inland Northwest Yucatan, Mexico.” *American Journal of Physical Anthropology* 154 (1): 140–51. https://doi.org/10.1002/ajpa.22490.

Standen, V. G., and B. T. Arriaza. 2000. “Trauma in the Preceramic Coastal Populations of Northern Chile: Violence or Occupational Hazards?” *American Journal of Physical Anthropology* 112 (2): 239–49. https://doi.org/10.1002/(SICI)1096-8644(2000)112:2<239::AID-AJPA9>3.0.CO;2-3.

Steadman, Dawnie Wolfe. 2008. “Warfare Related Trauma at Orendorf, a Middle Mississippian Site in West-Central Illinois.” *American Journal of Physical Anthropology* 136 (1): 51–64. https://doi.org/10.1002/ajpa.20778.

Strezewski, Michael. 2006. “Patterns of Interpersonal Violence at the Fisher Site.” *Midcontinental Journal of Archaeology* 31 (2): 249–79. https://doi.org/10.1179/mca.2006.012.

Tegtmeyer, C. E. 2017. “Violence in the Canyons: The Human Cost of Raiding and Warfare in Northeastern Arizona (AD 300-~ 1300).” Doctoral {{Dissertation}}, Department of Anthropology: University of Nevada, Las Vegas.

Torres-Rouff, Christina, and María Antonietta Costa Junqueira. 2006. “Interpersonal Violence in Prehistoric San Pedro de Atacama, Chile: Behavioral Implications of Environmental Stress.” *American Journal of Physical Anthropology* 130 (1): 60–70. https://doi.org/10.1002/ajpa.20315.

Tung, Tiffiny A. 2007. “Trauma and Violence in the Wari Empire of the Peruvian Andes: Warfare, Raids, and Ritual Fights.” *American Journal of Physical Anthropology* 133 (3). https://doi.org/10.1002/ajpa.20565.

Walker, Robert S., and Drew H. Bailey. 2013. “Body Counts in Lowland South American Violence.” *Evolution and Human Behavior* 34 (1): 29–34. https://doi.org/10.1016/j.evolhumbehav.2012.08.003.
