# Supplementary material for: Subsistence strategy mediates ecological drivers of human violence
Source: PLoS One. 2022 May 23;17(5):e0268257. doi: 10.1371/journal.pone.0268257 (PMC9126380; doi:10.1371/journal.pone.0268257)
Supplement: Attachment — Submitted filename: Response to Reviewers.pdf [file pone.0268257.s003.pdf]

January 3, 2021

PONE-D-21-31553
Subsistence strategy mediates ecological drivers of human violence
PLOS ONE

We would very much like to thank the reviewers for their incisive and valuable comments.

Our revised manuscript and the responses presented here focus on addressing the three main concerns expressed by the academic editor. Below, we provide a point-by-point response to reviewer comments. Where major in-text changes were made we highlight revised text with red font.

Academic editor's main points:
(1) Reviewer #1's requests for clarification
(2) the same reviewer's concern that environmental change complicates the assumption that modern estimates of NPP accurately reflect prehistoric ones.
(3) providing additional support for the assumption that skeletal trauma is a reliable measure of violence per se, a concern shared by both reviewers.
(4) The map figure copyright concern: the map used in our manuscript is an original creation by the authors and thus does not have any copyright violation.

Reviewer #1

Comment: Interpersonal violence and warfare are two very different cultural phenomenon and cannot be conflated. Wife-beating is interpersonal violence and it can be followed and explained in the archaeological and ethnographic records; however, wife-beating and organized, lethal warfare between armed groups have completely different causalities and trajectories.
Response: Great point regarding definitions. We clarify throughout the revised manuscript to focus on interpersonal (primarily inter-group) violence. However, we should note that intra- and inter-group violence tend to co-occur (Lutz 2007; McCool et al. 2020; Nordstrom, 1998; Tung, 2014), and that changes in intra-group violence should be affected by the process outlined in our theoretical setup. For example, resource scarcity may promote intra-group competition that increases interpersonal violence (Daly, 2017), and status and mating competition may intensify within group animosities that result in interpersonal violence (Chagnon, 2013).

Comment: To lump together all skeletal trauma and labeling it "violence" fails to distinguish accidental trauma (breaking one's leg from a fall) from the kind of systematic trauma arising out of warfare (forearm fractures, fontal bone fractures).
Response: We apologize for the confusion. Our archaeological dataset relies entirely on publications that explicitly provide trauma data that is attributable to interpersonal violence and excludes accidental trauma and post-mortem damage. We have revised our methods section to clarify.

Comment: The authors also fail to take into consideration chronological variability in the occurrence of violence/warfare. They conflate centuries or even millennia into single events,

without realizing that violence varies markedly over time in any given area. This fact, by itself, argues against their primary hypothesis that there is a strong causal correlation between violence/warfare and subsistence economy. People in one area, with a particular subsistence economy can experience a century of warfare, and many centuries without violence. There is no sense of chronological control in this study and no effort to look at when warfare occurs and when it fades away.
Response: We feel it is important to note that our primary result is not that there is "a strong causal correlation between violence/warfare and subsistence economy" (nor is it our "primary hypothesis"). It is the case with all multiregional archaeological analyses that chronological gaps will exist. Afterall, there is not a single region in which complete temporal resolution exists for data on violent trauma. Given this, we flatten temporal depth because we are interested in whether large physiographic regions differ in *average* rates of violence in response to variation in macroscale ecological conditions. Far from unusual, averaging diachronic variability is standard practice for multiregional studies in bioarchaeology (e.g., Allen et al. 2016; Arkush and Tung 2013 (flatten intraperiod variation); Beier et al. 2020; Delgado-Darias 2017; Fibiger et al. 2013; Kohler et al. 2014; Milella et al. 2020; Mummert et al. 2011; Pomeroy and Stock 2012; Scott and Buckley 2010:512; Standen et al. 2020; Steadman 2008:59; Vercellotti et al. 2014; Zhang et al. 2020), particularly in areas with limited radiocarbon datasets.

Comment: To simply impute warfare to skeletal trauma for a particular world area represents a basic misunderstanding of the data.
Response: As stated in the methods section, the archaeological dataset is a measure of generalized interpersonal violence, not warfare. Our revised methods section also points out that our dataset does not include all skeletal trauma, only violent trauma. Because rates of intra-group violence tend to be low in small-scale societies (Bohem 1999; Wrangham et al. 2006) and co-occur with inter-group violence (Nordstrom 1998; McCool et al. 2020; Lutz 2007; Tung 2014) – see revised intro and methods sections – changes in rates of population-level generalized violence will largely be attributable to fluctuations in rates of *inter-group* violence, which should co-occur with intra-group conflict. However, as stated above, changes in intra-group violence should be affected by the process outlined in our theoretical setup. Our main goal with this paper is to assess how macroscale ecological conditions impact generalized rates of interpersonal violence, and whether subsistence economy has a mediating effect.

Comment: In an attempt to push an incredibly complex and diverse global data base into their hypothetical models, they have papered over so much variability as to make their conclusions irrelevant. I cannot finish this review without commenting that is singularly the most jargon-laden, almost incomprehensible paper on this topic that I have encountered. Had the authors given more consideration into making their arguments coherent rather than technical, they might have identified some of their basic flaws.
Response: We contend that a key strength of our dataset is that it is "complex and diverse". It is not possible to address the reviewer's concern that "they have papered over so much variability as to make their conclusions irrelevant" without additional details regarding how the complexity and diversity of our dataset renders our model results (from which our conclusions follow) "irrelevant." As for the comment regarding the language and clarity of our paper, it is fully in-line with the broader literature on the evolutionary and ecological drivers of human violence (e.g., Allen et al. 2016; Bowles 2009; Glowacki et al. 2017; Zefferman and Mathew 2015; etc.).

We suggest that the "incomprehensibility" may stem more from divergent research traditions between the authors and reviewer, rather than incoherent writing.

Reviewer #2

Comment: Line 47: I don't think 'career' is a good word here; you could use 'behaviour' or 'social behaviour'
Response: Changed to 'behavior.'

Comment: This section is a bit too concise, which negatively affects clarity, and it leaves several questions unanswered. A lot of work done on violence in ancestral human societies has used data on different geographic areas and cultures, including work you cite (e.g. Bowles 2009), so it is not clear why and how your cross-cultural approach is novel.
Response: We have expanded the introduction, included new citations, and worked to clarify our setup with more explicit theoretical expectations. While our primary focus is on the adaptive payoffs of interpersonal and inter-group violence, our revised introduction includes content recognizing some of the broader research efforts, including: "Recent research also explores the coevolution of inter-group violence with intra-group cooperation, altruism, and the emergence of complex social systems (Bowles 2009; Rusch 2014; Zefferman and Mathew 2015)."

We agree that a lot of excellent work has been done on cross-cultural approaches to human violence. However, the majority of the formal cross-cultural research focuses on (1) the co-evolution of coalitional violence with cooperation, parochial altruism, and cultural group selection (e.g., Bowles 2009), (2) the categories of available rewards for participation (e.g., Glowacki and Wrangham 2013), and (3) punishing free-riders (e.g., Mathew and Boyd 2011). While these studies are vitally important, our study provides a novel perspective by seeking to explain how variation in large-scale ecological and economic conditions structure the social and material payoffs for inter-group (and interpersonal) violence. To our knowledge, there is no published research evaluating how macroscale resource conditions and modes of subsistence affect the adaptive payoffs for violence, especially on the scale presented in our research. Our findings: that violence among foragers and horticulturalists peak in productive, homogeneous environments, while violence among farmers peaks in marginal, heterogeneous environments, are novel and have implications for the evolution of interpersonal violence and warfare.

Comment: You also don't cite some seminal work on the topic, including on the role of culture, for example Zefferman & Mathew 2005.
Response: Thank you for clueing us into this literature. This reference and several like it have been included in our revised introduction.

Comment: Line 67: It would be useful to have a definition of violence at the start of the introduction. Do you use the term for all sorts of violent-like behaviours, including bullying and passive aggression, or it's only for potentially lethal violence?
Response: We clarify throughout to focus on inter-group violence (warfare, raids, feuds) and to a lesser extent general interpersonal violence, although much of the processes outlined in the paper should impact both intra- and inter-group violence (e.g., status and mating competition).

Comment: Lines 80-82: I think you should mention here that some studies have not found a

positive relationship between participation to war and adaptive benefits (e.g. Ferguson 1989; Beckerman et al 2009).
Response: We include Beckerman et al. 2009 in the revised introduction to show that debate persists. We do not include the Ferguson 1989 paper as Chagnon 1989 provides a robust refutation of Ferguson's claims.

Comment: Lines 83-88: I don't fully get your point here. You say you want to focus on cross-cultural variation and not on the payoffs of violence, but then your aim is to analyse the ecological and economic conditions triggering violence. Ecological and economic conditions are clearly linked to payoffs, so I don't understand the distinction you are trying to make. Moreover, ecological and cultural factors are linked together; for example, a group living in a highly seasonal environment may be forced to fight with other groups during periods of food scarcity and also develop group norms that reward warriors, both factors affecting payoff. I think this section should be clarified to make your point stronger.
Response: We have expanded our theoretical setup to provide greater clarity and set out more explicit expectations. As suggested by the reviewer, resource violence and social rewards may very well be linked, and we address this possibility in the revised introduction and discussion sections. However, many evolutionary anthropologists studying violence find resource procurement to be an irrelevant or epiphenomenal motivation (e.g., Chagnon 2013; Glowacki and Wrangham 2013; Kelly 2005; Macfarlan *et al.* 2018), instead positing that direct access to social rewards (i.e., status, mating, alliances) provide the key indirect fitness payoffs. Alternatively, many scholars studying inter-group violence propose that the primary motivation for violence is the procurement or defense of scarce, monopolizable resources (e.g., Allen et al. 2016; Ember and Ember, 1992; Field, 2008; LeBlanc 1999; Vayda, 1976), with social rewards taking an epiphenomenal position.

We propose to test these hypotheses by noting that each hypothesis implies a different functional relationship between violent behavior and socioecological conditions. If the resource procurement hypothesis is correct (if the payoffs to violence are primarily material resources), then violence should positively co-vary with a scarce and variable resource distribution. If the social rewards hypothesis is correct (if the payoffs to violence are social rewards), then violence should positively covary with an abundant and predictable resource distribution. We recognize in the revised introduction that, as the reviewer states, fighting over resources can be linked to social rewards, but contend that in this case resource violence would only be initiated with resource scarcity, and would diminish as resources become increasingly abundant and predictable. In addition to this basic test, our analysis offers a novel avenue for the assessment of resource violence, in particular whether it yields increasingly high payoffs with the emergence of sedentism, storage, privatization, and wealth. We summarize these divergent expectations with a new figure (Fig 1).

Comment: Line 107: it would be useful to explain in details how your dataset represent an expansion of previous ones. Is this in relation to societies included, precision of the data, range of variables considered or else?
Response: We added: "The dataset developed here builds off prior studies by providing additional archaeological and ethnographic samples and improves precision by excluding archaeological groups with small sample sizes."

Comment: Lines 132-134: you should discuss here whether/to what extent you can reliably say that these signs of trauma are due to pre-/peri-mortem violence or to post-mortem rituals. There has been a lot of debate whether traumas on bones are really reliable indicators of violence; e.g. see Fry's book (2013).
Response: Thank you for this suggestion. The revised methods section states that bioarchaeologists have robust methods for differentiating these types of trauma. For each of our archaeological samples only violent skeletal trauma is included, with post-mortem damage/rituals and accidents being excluded.

Comment: Line 136: you should give the definition of NPP here, the first time you introduce this acronym.
Response: This has been fixed.

Comment: Lines 136-138: if this is a recommendation for future studies, it would be better placed in the conclusions
Response: We include this caveat in the methods section as a preemptive response to readers who may be wondering why our samples are clustered in certain world regions. This clustering is due to the paucity of existing data rather than our own oversight. We include an additional sentence in the revised conclusion section calling for additional data in more diverse ecological settings.

Comment: Line 163: how far back in time are the archaeological data from? These has been a lot of environmental changes between prehistoric times and now, and if we also include global warming I don't think NPP data collected between 2000 and 2015 is a reliable measure of habitat productivity to be linked to violence occurring thousands (or even hundreds) of years before. You claim that "While NPP provides a measure of modern productivity, the relative NPP ranking of each society should have remained consistent over time due to their broad geographic range representing general physiographic regions rather than variants within single ecological or climatological regimes", but this sounds a weak claim to me. Do you have any evidence to support this claim? is there any reference to previous work that has tested this assumption? There have been both micro-and macro-climatic changes at the end of the Holocene, that have been suggested to lead to changes in social organisation and the emergence of agriculture (e.g. Shennan 2018), so this point is really critical for the reliability of your findings.
Response: To address this point, we added the following content to the revised methods section: "Modern NPP has been used to predict numerous prehistoric phenomena including population density (Bradshaw et al. 2019; Eriksson et al. 2012; Timmermann and Friedrich 2016), habitat colonization (Codding and Jones 2013), resource scarcity (Allen et al. 2016) and more. While NPP provides a measure of modern productivity, the *relative* NPP ranking of each region should have remained consistent over time due to their broad geographic range representing general physiographic regions (i.e., bioregions) rather than variants within single ecosystems (see, Bioregions 2020). To better illustrate this point, we take as an example two regions included in our sample: the North American Colorado Plateau (CP) and the Illinois river valley (IRV). While the absolute NPP of these two regions certainly fluctuated throughout the centuries, there was no point during which the "mountain grassland and scrublands" of the CP were more environmentally productive (higher NPP) than the "tropical and subtropical dry broadleaf

forests” of the IRV (Bioregions 2020; Dinerstein et al. 2017). The *relative* differences in environmental productivity between these two regions are due to their differing physiographic characteristics resulting in very different ecologies, which have remained comparatively consistent for millennia. The same can be said about the relative variation in NPP between the other bioregions compared in our analysis and the observations clustered therein (e.g., the lowland neotropics of the Amazon Basin versus the Canadian boreal forests). At most, changes in absolute NPP would only serve to exaggerate or flatten the modeled curves, but would not lead to trend reversals.”

Comment: Line 181: it would be useful to add a binary control variable, at least in the preliminary analyses, to see if you find any difference between archaeological and ethnographic data, otherwise you cannot really disentangle whether difference between type of societies are real or due to the fact that different data are available for different societies
Response: We include a binomial model in the supplement that evaluates data type. We also include a new “Data Compatibility” section in the revised methods that works to address this concern and others mentioned below. In the revised introduction and methods section we discuss the need to used mixed archaeology/ethnography dataset due to inherent limitations in the available violence data.

Comment: Line 191: I believe you should compare a null model (containing no predictor variables) with your two base and full models. If there is no significant improvement from the null model, you should make no claims that NPP affects violence.
Response: In our revised manuscript we generate a null model and conduct a likelihood ratio test comparing the null model to the base model and the full model to the base model. The base model results in a significant improvement from the null model ($X^2 = 0.47$, $p = 0.0458$), while the MoS (full) model results in a significant improvement from the base model ($X^2 = 2.13$, $p < 0.0001$). More details are available in the revised manuscript and Supplement 2.

Comment: I don't understand from this section or from the methods, whether you have data on MoS for the archaeological and ethnographic data or just for the latter. Why not analysing the two datasets separately, since you say above that there are differences in how they can estimate violence?
Response: We apologize for the confusion. We categorized all samples by MoS and include an additional sentence at the beginning of the revised methods section to clarify. We do not analyze the datasets separately because, due to differential research traditions, ethnographic data is restricted to forager and horticultural groups while archaeological data almost entirely focus on agricultural groups. Thus, the ethnographic dataset would not have sufficient sample size to test the agricultural MoS while the archaeology dataset would have the same problem for foragers and horticulturalists. Thus, the combined approach taken here is necessary until ethnographic violence data is obtained for agriculturalists (especially non-western, non-state societies), and archaeological violent trauma data is generated for forager and horticultural societies.

Comment: Your results are in line with what one would predict based on previous work in behavioural ecology. However, I cannot see any discussion about the potential bias in the findings, introduced by combining together archaeological and ethnographic data, and on how

reliable NPP is to test your predictions. These are key aspects that would need to be addressed or at least highlighted to the reader to help a more critical evaluation of your findings.
Response: We included additional content in the revised discussion and conclusion sections regarding the combining of archaeological and ethnographic datasets. We acknowledge that differing data types may affect our models. However, until better datasets are available (see revised methods section), the approach taken here constitutes the best practice available, and our results follow strongly from theoretical expectations. We expanded our conclusion section to include these caveats and how we propose to treat them. We also included additional content in the methods about the use of relative NPP values and physiographic characteristics.
