# Supplementary material for: Subsistence strategy mediates ecological drivers of human violence
Source: PLoS One. 2022 May 23;17(5):e0268257. doi: 10.1371/journal.pone.0268257 (PMC9126380; doi:10.1371/journal.pone.0268257)
Supplement: Attachment — Submitted filename: Response to Reviewers.docx [file pone.0268257.s004.docx]

We would like to thank the editor and reviewers for their insightful commentary. Below, we provide a point-by-point response to review comments.

Reviewer comments:

**Reviewer #2**

Comment: Line 55, "We argue that the trend reversal between foragers and farmers": here I think farmers refer to agriculturalists, but at line 53 you use the term 'forager/farmers' for horticulturalists, so I am not sure where you draw the line between foragers and farmers in this sentence.

Response: We edited this sentence to refer specifically to agriculturalists.

Comment: Lines 55-60: I am ok with your argument, but it doesn't clearly follow from your finding that resource availability affects differently foragers and farmers. For example, why would farmers acquire "material resources to be transformed into social payoffs" only or more in unproductive environments?

Response: Farmers should acquire fitness-linked resource in all environments, but where resources are scarce and unevenly distributed individuals will receive higher relative payoffs for using violence to gain resources. Those with abundant resources should be more tolerant of theft and less willing (relative to those in poor environments) to use risk-prone strategies such as violence to obtain resources. As such, we don’t argue that farmers use violence to obtain resources only in poor environments, but that the payoffs for violence will be higher relative to those living in productive, homogeneous environments. We provide more detail on these relative payoffs using marginal utility theory on lines 365 – 374.

Comment: Line 73: I really don't think you can use data on chimpanzees to make a claim about all non-human animals. If you look at the literature on other species, you will find that lethal violence is probably way more common in humans in comparison to the majority of other primates and mammals.
Response: We agree and find that this sentence is distracting and have thus deleted it to save this point of discussion for later in the manuscript.

Comment: Line 276: it should read "while FOR farmers VIOLENCE negatively co-vary with productivity and homogeneity"

Response: This sentence has been fixed.

Line 321 "directly elevate inclusive fitness." I think there should be some refs at the end of this sentence

Response: We have added four references that support this statement.

**Reviewer #3**

I have only a few suggestions for clarification on some points, but also a recommendation for what would be a more major undertaking (dividing the foraging sample, which might require a larger sample size), and so therefore it is not required but might be considered for future.

Comment: 80/ Not sure that all would agree that those seeing inter-group conflict as rare/maladaptive have been effectively countered, though the authors do acknowledge that this debate continues.

Response: Fair point. Our objective with this statement is to acknowledge that important debate continues, but that debate is not the focus of the present research. We added additional text to emphasize that the ‘effective counters’ are for most, but not all, cases.

Comment: Odd to rely on the ethnographic record alone for foragers and horticulturalists when there is considerable archaeological evidence that might be drawn upon, though it is admittedly patchy. Some of the previous studies cited do drawn upon the archaeological record for foragers.
Response: We do include several archaeological samples for horticulturalists, but these data are indeed rare. As for foragers, most of the data, while extremely valuable, are highly localized and often suffer from exceedingly small samples sizes that are below our sample size cutoff (lines 163-164). We are in the process, over the next several years, of putting together a much larger archaeo-ethnographic database which will expand upon the present database and will hopefully provide a more comprehensive archaeological database on foragers. This does assume however that the recent increase in bioarchaeology studies on violence among small scale societies continues. For the time being however, we must rely on the limited information available and look forward to the production and release of additional datasets. We hope with the publication of this manuscript to encourage future work generating trauma datasets.

Comment: 159/ “This database operationalizes sigma by defining it as the number of individual skeletons with evidence of violent trauma divided by the total skeletal sample for each archaeological observation”; repeated line 187
¬ Surely this defines prevalence rather than sigma (a measure of variability), though from ln 258 it is being used here as a measure of the ‘rate of violence’?

Response: We changed “sigma” to “PV” for “proportional violence” and changed “rate of violence” to “proportion of violence” throughout.

Comment: 317/ “Ethnographers report evidence showing adult males frequently fight over mating opportunities and real or perceived infidelities.”
¬ This seems to contradict the previous statement regarding the low incidence of in-group violence conflict, assuming that opportunities for such infidelities are likely to be mainly within the group

Response: We feel this is an important point to make, as intra-group hostilities do certainly occur, even if the rates are much lower relative to inter-group rates. Our point here is that when mating rewards are at stake, motives for inter-personal violence should be consistent regardless of whether antagonists are within group or outsiders.

Comment: 345/ “Seeming exceptions prove the rule: in prehistoric California, delayed-return hunter-gatherers who rely on the storage of privatized resources follow the violence pattern observed here among agriculturalists.”
¬ So this is not taken into account in the analysis, and the ‘foraging’ mode of subsistence includes all h-g? Not clear how this follows the violence pattern seen among agriculturalists as stated, as h-g practicing storage are often in relatively rich environments (such as those of CA and the NWC), whereas the discussion here focussed on the increased violence among agriculturalists being predicted by more marginal/unpredictable environments. This seems a discrepancy rather than an accord.

Response: This an excellent point about CA and NWC in relation to environmental productivity and conflict. Unfortunately, we cannot further investigate empirically how these two areas fit into our models for two reasons: First, the NWC does not contain sufficient violent trauma data to be included in our models; Second, the Central California bioarchaeological database does not contain a measure for generalized violent trauma (which is how violence is measured for all of our archaeology samples), as the database divides all individual traumas into either blunt or sharp force. As such, individuals could potentially be double counted if they exhibit both sharp and blunt force trauma. We cite the CA study because these largely delayed-return foragers follow a pattern similar to the farmers in our model. While the Central CA coast is indeed productive, we would need to model this relationship to assess where they fit in along the NPP and violence spectra, which we cannot, unfortunately, do.
¬ Why not divide foragers? The division between immediate- and delayed-return h-g is well established and in many ways it is odder to lump them than to divide them.

Response: We completely agree that this analysis could be improved by converting categorial subsistence economies to a continuous scale from immediate- to delayed-return. However, we should note that the foraging societies included in our database are immediate-return, and have revised the text to include this information (line 127). For the present analysis, the pool of available data is simply not large enough to further divide these data into more fine-grained economic categories, especially in relation to model sample sizes. For now, we are specifying the best possible models with the given data, which we hope will improve in size in quality in the coming years. We hope our research here will encourage future research on prehistoric violent trauma, particularly among foragers and pastoralists.

Comment: 382/ “First, if survivorship is frequently in jeopardy one should see far greater demographic parsimony regarding participation in resource conflict”
¬ Not clear that this follows, as there is little point in all and sundry engaging in violence (children, the elderly?) when they are more likely to be a hindrance than help to those better suited to violent conflict through both age and skills (e.g., hunting).

Response: Fair point. We do not mean to suggest that demographic participation should be universal if violence functioned to prevent starvation, but merely to state that participation should be much broader than the typical pattern of young adult males. Certainly, if basic survivorship were in jeopardy and violence was the only means of somatic maintenance we should expect far greater sex-based parity in participation, yet this does not commonly occur. The point we make is simply that the common pattern of young adult male organization and participation in collective violence heavily implies motives other than basic somatic maintenance in the face of starvation. We added a statement to clarify that a key point is the notable absence of sex-based parity in participation.
